# Flexoelectricity in Biological Materials and Its Potential Applications in Biomedical Research

**DOI:** 10.3390/bioengineering12060579

**Published:** 2025-05-28

**Authors:** Melika Mohammadkhah, Vukasin Slavkovic, Sandra Klinge

**Affiliations:** 1 Technische Universität Berlin, Institute of Mechanics, Chair of Structural Mechanics and Analysis, Straße des 17. Juni 135, 10623 Berlin, Germany; 2 Department of Applied Mechanics and Automatic Control, Faculty of Engineering, University of Kragujevac, Sestre Janjić Street, Nr. 6, 34000 Kragujevac, Serbia

**Keywords:** flexoelectricity, piezoelectricity, biological materials, cell membranes, hearing mechanism, bone remodeling, tissue engineering, electromechanical behavior

## Abstract

Flexoelectricity arises in materials under strain gradients, which can be particularly significant for situations in which the existence of other electromechanical properties is absent or generating large flexoelectric properties is achievable. This effect has also been observed in some biological materials, whose understanding can hugely help to further enhance our understanding of vital biological processes like mechanotransduction, as well as the development of applications in regenerative medicine and drug delivery. While the field of flexoelectricity as a relevant topic in biological materials is relatively new and still developing, the current study aims to review available results on flexoelectric effects in biological materials such as cells and cell membranes, hearing mechanisms, and bone, and their potential applications in biomedical research. Therefore, we first provide a brief background on two main electromechanical couplings (piezoelectricity and flexoelectricity) and further, how flexoelectricity has been experimentally and theoretically identified. We then review flexoelectricity in different biological materials as the main aim of the current study. Within that, we provide additional emphasis on the influence of this effect on bone and bone remodeling. In particular, the study outlines current limitations and provides potential directions for future work, emphasizing the crucial role in the development of next-generation electromechanical devices and optimizing their function in the area of biomedical research.

## 1. Introduction

Intrinsic (piezoelectricity and flexoelectricity) and extrinsic (electrochemical and electrostatic effects) electromechanical coupling exist in a wide diversity of materials, including man-made and natural materials [1,2]. Understanding the underlying principles of these couplings is essential for both the innovation of next-generation electromechanical systems and the interpretation of their functional behavior. Accumulations of electric charge in response to applied mechanical loading/deformation have been employed as building blocks for devices like actuators [3], sensors [4,5], photoflexoelectricity [6], energy harvesting devices [7,8,9], structural health monitoring [10], and in Triboelectricity [11]. Among several electromechanical effects, flexoelectricity, as the focus of the current study, has been observed in biological systems [12], important biological functions such as auditory sensing [13] and swelling of neurons associated with action potentials [14].

One of the most intriguing applications of flexoelectricity is the creation of materials with piezoelectric-like properties, without relying on traditional piezoelectric materials [15]. Recently, there has been a surge of attention towards flexoelectricity, resulting in numerous reviews on the concept across various fields, including physics and mechanics, materials science, and nanotechnology [16,17,18,19,20]. However, the focus on the existence and influence of this effect on biological research is sparse. Therefore, the current study aims to review the existing studies that have investigated flexoelectricity in biological materials since such critical knowledge will have a key role in recent and ongoing advancements in mechanotransduction in biological tissues, modern therapeutic potential such as tissue engineering, regenerative medicine, and drug delivery. The current work is structured as follows: a comparison between piezoelectricity and flexoelectricity as the main intrinsic electromechanical couplings is first provided in Section 2 to deliver sufficient background on the topic. Section 3 discusses how flexoelectricity is experimentally and theoretically identified in materials. In Section 4, the emphasis of this review turns towards the flexoelectric effects in biological materials, namely, cells, bio-membranes, hearing mechanisms, and bone materials. Section 5 explores the potential biomedical applications of flexoelectricity along with recent advances in the area. Section 6 discusses the environmental factors influencing piezoelectricity, as well as key experimental and theoretical challenges that could impede further progress in the field. Finally, Section 7 concludes the paper by outlining the future research directions in this field.

## 2. Flexoelectricity vs. Piezoelectricity

**Figure 1:**
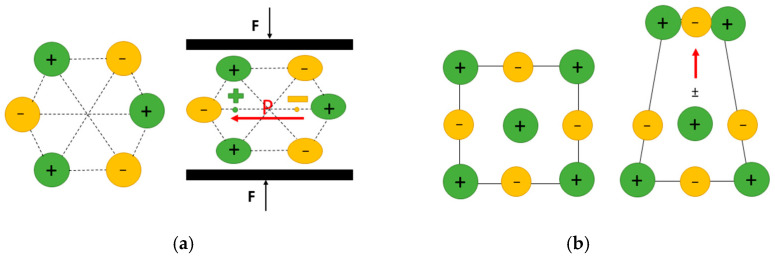
(**a**) Schematic illustration of direct piezoelectric effect: applied mechanical loading (F) causes a displacement of the positive and negative charge centers, resulting in a change in polarization (P) and generating an effective electrical field. (**b**) Schematic illustration of the flexoelectric effect: due to inhomogeneous strain, the centers of the positive and negative charges shift, creating a dipole moment opposite to the strain gradient, which produces a non-zero polarization (red arrow).

Piezoelectricity: Piezoelectric materials represent an important class of smart materials; they become electrically polarized under mechanical stress and, conversely, undergo strain in response to an applied electric field, with the deformation proportional to the field strength. Piezoelectricity is the linear coupling between strain and electric field (polarization) as stated in Equation (1) and requires a noncentrosymmetric arrangement (lack of inversion symmetry) of dipole moments within a material (Figure 1a). Therefore, this remarkable property belongs to a rather small subset of insulating materials [2].

(1) $$P_{i}=\epsilon_{ijk}\varepsilon_{jk},$$

where $P_{i}$ is the polarisation, $\epsilon_{ijk}$ is the piezoelectric strain tensor and $\varepsilon_{jk}$ is the strain tensor [21].

Piezoelectricity has been thoroughly investigated in bulk and mesoscopic materials and more recently has gained significant interest in nanomaterials [17,22,23]. Although piezoelectricity is well-established and widely utilized, its presence is limited to materials with non-centrosymmetric crystal structures and only below their Curie temperature. To address this limitation, the emerging concept of using flexoelectricity to induce effective piezoelectric behavior in non-piezoelectric materials has become increasingly appealing [24,25].

Flexoelectricity: Flexoelectricity, an alternative electromechanical effect that has gathered relatively less attention, involves the generation of electrical polarization by a strain gradient (direct effect) or the induction of mechanical strain by an electric field gradient (converse effect). Mathematically, the flexoelectric effect is a second-order effect as stated in Equation (2). In solid ionic crystals, flexoelectricity arises from the non-uniform displacement of ions under a strain gradient (inhomogeneous strain), which breaks inversion symmetry and generates a net polarization within the crystal (Figure 1b). Therefore, in contrast to piezoelectricity, flexoelectricity is present in any crystalline material [2]. The absence of symmetry constraint makes the flexoelectric materials suitable for most cases where non-uniform electric field distribution and non-uniform strain distribution exist [20]. In most dielectric materials, flexoelectricity is known to contribute only modestly to polarization, with typical flexoelectric coefficients ranging from $10^{-10}$ to $10^{-8}\;{cm}^{-1}$ [26]. However, recent studies have shown a significant enhancement of flexoelectric polarization in nanomaterials lacking inversion symmetry, where the flexoelectric coefficient can reach values as high as $10^{-8}$ to $10^{-4\;}{cm}^{-1}$ [27,28]. Flexoelectricity has also been observed in liquid crystals and semiconductors (not confined to dielectric materials). While it is considered a higher-order electromechanical effect compared to piezoelectricity [29], its contribution is generally smaller at macroscopic scales. However, at the nanoscale, where large strain gradients can be more easily generated, flexoelectric effects become more pronounced. Materials with unique geometries that allow for significant strain gradients tend to exhibit more noticeable flexoelectric behavior [17].

(2) $$P_{i}=\mu_{ijkl}\frac{{\partial \varepsilon }_{jk}}{{\partial x}_{l}},$$

where, $\mu_{ijkl}\;$ is the flexoelectric coefficient, $\frac{{\partial \varepsilon }_{jk}}{{\partial x}_{l}}\;$ is the elastic strain gradient [21].

In the 1960s, the concept of flexoelectricity was first introduced by Kogan [21]. However, research on its manifestation in solid materials remained sparse for many years, with most studies concentrated on liquid crystals [30]. This changed in the early 2000s, when systematic experimental studies on flexoelectricity in ferroelectric ceramics revealed that the response could be several orders of magnitude stronger than theoretical estimates had predicted [31]. Additionally, as length scales decrease, larger strain gradients—and consequently stronger flexoelectric effects—are expected. Historically, flexoelectricity was regarded as a high-order effect of piezoelectricity [21], but subsequent research has shown that it extends beyond that [26]. First, flexoelectricity is present in all dielectrics, which reduces our reliance on piezoelectric materials. Second, flexoelectricity is an intrinsic effect, with strength increasing as sample size decreases [21,31]. This characteristic opens up the possibility of enhancing the flexoelectric effect in common dielectrics [25] and suggests the potential to engineer composites with far stronger apparent piezoelectricity. In this regard, flexoelectricity not only holds promise for advancing nano-device development [32] but also offers an alternative to the continuous search for new materials when stronger electromechanical coupling is required [33].

## 3. Experimental and Theoretical Identifications of Flexoelectricity

The key consideration in experimental identification of flexoelectricity is how to produce sufficiently large strain gradients so that the phenomenon can be relatively noticeable. Two different experimental approaches, (i) direct macroscopic characterization and (ii) indirect microscopic characterization, can be used to evaluate flexoelectricity in solids. Macroscopic identification is typically performed using a variation of the two common methods of (i) bending a cantilever beam or thin film [34,35] and (ii) uniaxial compression of the truncated pyramid or cone-shaped material [31,36]. In the indirect approach, the flexoelectric coefficient can be extracted from various physical phenomena closely related to flexoelectricity, but the analysis approach differs from current or electric polarization measurements namely piezoresponse force microscopy (PFM), Second Harmonic Generation (SHG) Analysis, electric field microscopy, optical methods (interferometry, ellipsometry) etc. [37,38,39]. The review article by Kołodziej et al. (2024) [2] has comprehensively covered the details of several experimental methods, that is recommended to interested readers.

Measurement of flexoelectric coefficients: The bending curvature of a beam results from the gradient of its longitudinal normal strain in the thickness direction, caused by the flexoelectric effect. Careful measurement of the curvature and the induced electrical output leads to obtaining a component of the flexoelectric coefficient ($\mu_{1122}$) of the material [40]. Regarding other components of the flexoelectric coefficient, the compression of a truncated pyramid was proposed to create a strain gradient in materials. Due to their different areas, the stress generated differs at the top and bottom surfaces of the truncated pyramid, producing a longitudinal strain gradient and thus a flexoelectric polarization. Table 1 summarizes measured flexoelectric coefficient values for various flexoelectric materials with their measurement techniques. Needless to emphasize that the sample should also be as thin or small as possible for these methods to gain a higher flexoelectric effect. However, in bulk materials, (i) the concept of introducing small defects to locally trigger the flexoelectric effect has been proposed [41]. After uniform load application, there would be a large strain gradient locally developed around the defect (around the corner of a micro-hole) due to stress concentration. (ii) Large strain gradients can still be generated without introducing micro-defects, using elastic waves. As an elastic wave propagates through a dielectric material, it creates a time-varying strain gradient and its spatial gradient. The magnitude of this strain gradient depends on the amplitude and wavelength of the elastic wave, allowing for the generation of large strain gradients in bulk dielectrics through acoustic waves [16].

Studies on flexoelectricity have not only focused on experimental work but have also led to significant theoretical advancements across various materials. The size effect of flexoelectricity was initially proposed through theoretical predictions, rather than experimental findings [25]. Fundamentals of flexoelectricity in solids [26] and the theory of flexoelectricity on a piezoelectric film [55] have been well described in the literature. The history of development of theoretical studies of flexoelectricity in materials from the late 1950s up to more recent theoretical calculation methods, such as the finite element method, phase-field modelling, density functional theory, and local maximum-entropy meshfree methods, is also well reviewed [16,19,56] to explore the direct and converse flexoelectric effect. Currently, the term ‘flexoelectricity’ is used in two areas of soft matter (liquid crystals and biological materials) [13,57,58] and common solids such as non-piezoelectric hard materials [33,59]. In soft biomaterials, flexoelectricity is best interpreted using a liquid crystal model [60]. The effect manifests as curvature-induced polarization, which results from the rotation of irregularly shaped polarized molecules under splay or bent deformations.

Theoretical studies on flexoelectricity fall into two main categories: atomistic and continuum approaches. Molecular dynamics (MD) simulations—based on either the rigid ion assumption [61,62] or the core-shell model [25]—have also been employed to study flexoelectric effects in finite-sized samples. The lattice-dynamical approach has been used to explain the origin of flexoelectricity in certain cubic crystalline ionic salts, pervoskite dielectrics, and some semiconductors [63]. Stengel, using density-functional perturbation theory, proposed a more comprehensive model that includes both electronic and ionic contributions to bulk flexoelectricity [64]. For amorphous systems, Schulz and Marvan developed a microscopic chain dynamics model to explain flexoelectricity in glassy polymers [65]. By relating local deformation to local vectors, they derived a flexoelectric coefficient composed of three components: gradients of local shape change, local volume change, and local rotation. Building on this, Marvan and Havránek proposed that the rotation of frozen dipoles in polymers is governed by the free volume available to each dipole. They derived an expression showing that the flexoelectric coefficient is proportional to both the number and magnitude of these dipoles [66]. In continuum-level modeling, one of the major challenges lies in accurately determining input parameters, which are typically obtained from either experimental measurements [31] or atomistic simulations [61]. However, significant discrepancies—often by orders of magnitude—exist between these two methods. This highlights the need for multiscale modeling to achieve a deeper understanding of flexoelectric phenomena. Since the focus of the current study is on the existence and influence of flexoelectric effects in biological materials, the interested reader is referred to the most recent reviews for a detailed description of the theory of flexoelectricity [16,19,67].

## 4. Flexoelectricity in Biological Materials

Several biological materials, such as cellulose- and protein-based materials, bones, viruses, cells, and bio-membranes, have a remarkable flexoelectric response [1,57,68,69,70,71,72]. The phenomena such as the fracture and repair of bones [73,74], the hearing mechanism [75], and electromechanical properties of cell membranes [57] are all believed to closely relate to the flexoelectric effect. The first study of the flexoelectricity in biological materials could be traced back to 1975 by Williams and Breger [76], where some of the electromechanical properties of bones were considered to have likely originated from “gradient polarization”, but the mechanism was not clear at that time.

### 4.1. Flexoelectricity in Cells

Cell mechanics plays a crucial role in cellular development, function, metabolism, and the regulation of nuclear responses [77,78]. Understanding cell mechanics under electromechanical stimuli is crucial not only for various cellular functions but also for advancing modern medical therapies, including tissue engineering and regenerative medicine [79]. Thus, establishing a basis for accurately characterizing the electromechanical transduction within the biological cells will hugely advance our knowledge to capture the complex mechanisms at the cellular level.

The cell consists of the nucleus and the cytoplasm—a fluidic matrix that typically surrounds the nucleus and is enclosed by the cell membrane [80]. The cytoskeleton is made of different structural components such as microtubules, intermediate filaments, and actin filaments. Several theoretical, computational, and experimental studies have been reported to accurately quantify the cellular response to mechanical stimuli [77,79,81]. Biological molecules have demonstrated piezoelectric properties, making them valuable for medical applications from sensing to surgery [82]. In spite of the paucity of experimental data for the flexoelectricity of biomaterials in the literature, research is still ongoing to investigate the possible role of flexoelectricity in cells [80,82]. Singh et al. (2020) [82] proposed a coupled two-dimensional electromechanical model to investigate the behavior of biological cells incorporating piezoelectric and nonlocal flexoelectric properties of their organelles under external forces. Their findings suggest that flexoelectricity may be a dominant mechanism, generating electric fields up to four orders of magnitude stronger than those from piezoelectric effects alone. The model’s output was highly sensitive to variations in the flexoelectric coefficients. Notably, they observed that mechanical degradation of the cytoskeleton enhanced both piezoelectric and flexoelectric responses [82].

The separate electromechanical response of individual structural components (e.g., microtubules, due to their key functions in cell division, maintaining cell structure, and intracellular transport) has also been studied [83,84,85]. The regulation of microtubule dynamics is essential for proper cell function and the transport of various cellular components [86,87]. A recent two-dimensional electromechanical framework of the biological cell, including microtubules under distinct mechanical loading conditions originating from the external environment has been recently proposed [70], which revealed that the number of microtubules significantly impacts effective elastic and piezoelectric coefficients, affecting cell behavior based on shapes and sizes of organelles, structure, microtubule orientation, and mechanical stress direction. Since flexoelectricity is size-dependent, they demonstrated that its impact on the electromechanical model is significantly more pronounced than that of introducing piezoelectric coupling alone. The effect of flexoelectricity is highlighted by a new parameter, the maximum electric potential ratio, which depends on the orientation angle and shape of the microtubules. Flexoelectricity exerts a substantial influence on the biological cell, and the insight obtained from the electromechanical behavior of cells can advance medical therapies such as drug delivery systems, tissue engineering, and regenerative medicine.

### 4.2. Flexoelectricity in Bio-Membranes

Biological membranes are not purely mechanical entities and exhibit flexoelectric effects since they bend quite easily [88]. Flexoelectric effects are relevant to studying ion channels, thermal fluctuations, and the equilibrium shape of the vesicle [12,89,90,91]. Flexoelectricity in membranes results from the interaction between the electric field and the molecular multipoles constituting the membrane, leading to an inhomogeneous electric force across the membrane, as well as the ions bound to these molecules [92]. The microscopic groundwork of flexoelectricity was established by Petrov for biological membranes in a series of founding studies [57,58,93,94]. The main theoretical approaches to studying flexoelectricity in membranes rely on continuum models. Ambjörnsson et al. (2007) [95] developed a continuum model to describe the electromechanical effects of applying a static potential across the membrane [95] to assess the electrostatic contribution to membrane instability and bending rigidity using the Poisson-Boltzmann equation. They showed that the membrane became stiffer towards bending (positive effect) and experienced lower surface tension (negative effect) under an applied potential. In contrast to the continuum approach, Harland et al. (2010) proposed a model describing electromechanical coupling, voltage-dependent, discrete adsorption of ions to the phospholipid polar head groups [96]. Disregarding the discreteness of charges results in lower flexoelectric coefficients in comparison with experimental data [57]. However, the flexoelectric effect demonstrated by continuum models remained qualitatively acceptable.

Bio-membrane flexoelectricity has also been studied using droplet interface bilayer (DIB) technology [97,98]. In this approach, lipids serve as organic surfactants in oil–water emulsions to create biomimetic membranes at the intersection of neighboring droplets. When two droplets come into contact, the lipid monolayers adhere, forming a capacitor. The contact angle at the monolayer–bilayer meniscus is determined by the tension balance between the two interfaces. Notably, this technique can be used to investigate mechanotransduction and the interfacial properties of unsupported liquid biomimetic membranes. In bio-membranes, flexoelectricity manifests as curvature-induced polarization [58], meaning that bio-membranes with smaller radii (higher curvature) exhibit a stronger flexoelectric response. High-frequency membrane deformation was shown to be capable of producing a significant flexoelectric current, whose value is related to the curvature of the interfacial membrane, suggesting that flexoelectricity in bio-membrane can drive the activation of tension-gated channels within the membrane [99,100,101].

Later, Mozaffari et al. (2021) [88] attempted to investigate the flexoelectric role in modifying the entropic force between two fluctuating fluid membranes using a variational perturbative approximation to obtain a closed-form solution. Given that incorporating flexoelectricity complicates the already challenging problem of two purely mechanical fluctuating membranes, they proposed an approximate yet highly accurate closed-form solution for the entropic force between two fluctuating flexoelectric membranes. When flexoelectricity is considered, an enhanced attractive force was predicted for membranes that are close to each other, and an enhanced repulsion was reported when the membranes are further apart [88]. Although no detailed physical consequence of their calculation on biology was reported, we believe their modelling can be a promising approach in biological phenomena [88]. Galassi and Wilke (2021) [92] provided a detailed review on the coupling between mechanical properties and electrostatics in biological membranes with a key message that different effects mentioned in their work (effects of ions, PH effects, effects of external potentials etc.) are a simple physicochemical phenomenon in essence, but spatial and temporal interconnection of them occurring simultaneously and locally in different regions of the membrane is required to regulate very complex phenomena such as nerve impulse propagation and electromotility [92]. The influence of flexoelectricity is stronger in soft materials than in highly flexoelectric crystalline ferroelectrics, and the effect is significant at feature sizes on the order of microns [92]. Therefore, flexoelectric investigation of biological membranes holds the potential to further understand the complex cellular mechanism.

### 4.3. Flexoelectricity in Hearing Mechanism

Hair cells in the inner ear serve as the primary mechanotransducers, converting mechanical vibrations of sound into electrical action potentials that the brain interprets as auditory signals [102]. There are a bundle of 50 to 300 elongated microvilli known as stereocilia at the apex of each hair cell [13]. Both piezoelectricity and flexoelectricity contribute to membrane electromotility, with their magnitude and polarity depending on surface potential and membrane stiffness [71,92]. Several works have indicated that flexoelectricity is an important electromechanical coupling in the hearing system [103,104]. Specifically, there is now substantial evidence [13,75,102] to show that flexoelectricity is the major mechanism behind outer hair cell electromotility and impacts cochlear amplification.

Petrov and Usherwood (1994) [105] were the first to propose that direct flexoelectricity might trigger mechanotransduction in auditory hair cells by converting sound-induced changes in membrane curvature into displacement currents [105]. By a simple biophysical model, Breneman et al. (2009) [13] examined the reverse hypothesis to what Petrov and Usherwood (1994) [105] proposed, that changes in membrane potential induce flexoelectric driven stereocilia movements, meaning that flexoelectric stereocilia take electrical power entering the mechanoelectrical transduction channels and directly convert it into mechanical power in charge of amplification of sound induced vibrations in the inner ear [13]. Their model revealed that stereocilia are fast flexoelectric motors and highly efficient at capturing the energy in the extracellular electrochemical potential of the inner ear to generate mechanical power output. Their analysis explained the correlation between stereocilia height and the tonotopic organization of the hearing system (Tonotopic organization means that cells responsive to different frequencies are found in different regions at each level of the central auditory system, and that there is a standard relationship between this position and frequency. Shorter stereocilia are located in the high-frequency sensing region, and taller ones are located in the low-frequency sensing region).

Deng et al. (2019) [75] proposed a physical model of the mechanics and dynamics of hair bundles in the mammalian hearing mechanism by incorporating both membrane electromotility (flexoelectricity) and a physics-based nonlinear dynamical model. Their results showed that, due to flexoelectricity, certain combinations of inner surface charge density and membrane bending stiffness can cause hair bundle oscillations to become unstable, suggesting that this instability enables the amplification of weak acoustic signals. They also found that increasing the bending modulus or decreasing the magnitude of inner surface charge density can stabilize the system, potentially impairing the hair bundle’s ability to amplify external stimuli. Although the role of flexoelectricity in the hearing system has been noted before, their key finding was to show for the first time that flexoelectricity plays a key role in inducing the Hopf bifurcation state, which is believed to underlie several highly nonlinear aspects of the hearing mechanism [75].

### 4.4. Flexoelectricity in Bone

Bone is a hierarchical composite composed of an extracellular matrix (ECM) that includes an organic component—densely packed, aligned collagen fibers that provide flexibility—and an inorganic mineral phase, primarily crystallized hydroxyapatite (HA), along with osteogenic cells such as osteoblasts, osteoclasts, and mature osteocytes [106], which exhibit electromechanical properties. Bone is indeed piezoelectric [107]; the outer layer of cortical bone functions as a porous structure, featuring a piezoelectric solid framework saturated with a dielectric fluid. Piezoelectric property mainly originates from the nanocrystalline or liquid-crystalline ordered nature of complex ECM components, such as collagen [108]. Electroactive living cells typically make only a minimal contribution to the overall piezoelectricity of bone [107]. In addition to collagen’s piezoelectricity, streaming potentials—generated by fluid flow across charged surfaces—also contribute to the piezoelectric response in bone [109]. Mechanical deformation of bone tissue induces fluid flow through the canaliculi, causing ions to move along the channels. As these ions flow past oppositely charged canalicular walls, a potential difference is created between two points along the fluid stream [110]. New bone-forming cells (osteoblasts) were observed to accumulate on the surface of pure HA, which has neither collagen nor streaming ions. This suggests flexoelectricity as another electromechanical property mostly observed in bone mineral. Piezoelectric coefficients of HA were shown to be markedly weaker than the bone piezoelectricity (less than $0.001\;pC\;N^{-1}$) and comparable to decollagenized bone [111,112].

Bone’s hierarchical structure gives rise to various deformation gradients—such as internal porosity variations, elastic property differences, and piezoelectricity—that promote flexoelectricity, particularly near microcracks [113]. There used to be few and scattered studies providing evidence for flexoelectricity in bones. For example, Williams and Breger (1975) [76] suggested that certain electromechanical properties of bone might be explained by inhomogeneous piezoelectricity (gradient polarization). Later, Lakes (1980) [114] conducted a theoretical analysis on the potential role of strain gradients in bone, though the findings could not be confirmed due to the lack of quantitative data on bone’s flexoelectric coefficients. Fu (2010) informed bending-induced polarization in bones, mistakenly attributing this flexoelectric-like response to collagen [115]. Stock et al. (2011) [116] experimentally confirmed the presence of substantial strain gradient fields in bone. To clarify the origin of bone flexoelectricity, Vasquez-Sancho et al. (2018) [73] compared the flexoelectricity of bone and pure HA using the traditional cantilever system. The similarly reported values for flexoelectric coefficients of bone and HA, suggesting that HA flexoelectricity is the main source of bending-induced polarization in bone without needing to invoke collagen piezoelectricity (0.2 to 2.3 $nC\;m^{-1}$ for bone and 0.7 to 1.6 $nC\;m^{-1}$ for HA) [73]. At macroscopic scales, piezoelectricity governs the global response, while at smaller scales, flexoelectricity can be significantly stronger, dominating the local electromechanical behavior [115,117]. Using in silico modeling, Tiwari et al. (2021) [118] identified strain gradients as osteogenic stimuli and successfully predicted in vivo bone formation patterns as a function of these gradients.

Bone remodeling and crack healing: The process of bone remodeling has been described in detail in various studies [119,120,121]. Several Basic Multicellular Units (BMUs) are activated at the onset of a remodeling, originating from the central capillary of a Haversian or Volkmann’s canal near the remodeling site [122]. Osteoblasts, osteocytes and osteoclasts are involved in the physiological process of bone [123]. Osteocytes, embedded within the bone matrix, form an extensive communication network through gap junction–coupled cell processes and canaliculi. Bone remodeling is initiated by the damage or death of osteocytes, which release chemical signals that trigger the recruitment of osteoclasts at the leading edge of each BMU, marking the beginning of the resorption phase [120,124]. New bone formation is then initiated by osteoblasts by secreting unmineralized ECM, which later mineralizes to form mature bone tissue. Roughly 10–20% of osteoblasts differentiate into osteocytes—promoted by microcracks and mediated by release of various growth factors [125]—become embedded in the new bone matrix. The remaining cells either undergo apoptosis or become lining cells, which reside on the endosteal and periosteal surfaces and play a key role in transmitting biochemical signals during remodeling [126].

Following Fukada and Yasuda’s discovery of piezoelectricity in bone, bone remodeling has been closely linked to electromechanical phenomena [107]. Later, due to the discovery of flexoeletricity in HA, it was shown that bone can still generate electrical signals in response to mechanical stress—even in the absence of collagen—provided the deformation is inhomogeneous [73]. The recent discovery of bone flexoelectricity suggests that flexoelectricity near bone fracture sites may have physiological significance, where flexoelectricity is theoretically highest [74]. Microcracks, an inherent feature of bone, result from fatigue damage caused by cyclic stresses [127]. Flexoelectricity plays at least two different roles in bone remodeling in the vicinity of cracks, where there is neither collagen nor streaming potentials [73,112]: apoptotic triggering of the repair protocol i.e., apoptosis of the osteocytes near the crack [128], and electro-stimulating the bone-building activity of osteoblasts, i.e., increased maturation and mineralization of osteoblasts over the subsequent differentiation days [74]. Bone micro-fractures create mechanical inhomogeneities with large local strain gradients that can generate strong flexoelectric signals. HA can also generate signals that guide repairing cells. As the crack begins to heal, the apex shifts, acting as a signal to direct the flow of healing cells, thereby guiding osteoblast activity toward the damaged areas. However, the nature and origin of these signals remain unknown [117,129].

Experimental and computational models of bone remodeling using the flexoelectric effect: The experimental measurement of electromechanical properties of bone manifests in several aspects. The bending response of a bone cantilever differs from that of a collagen-free bone specimen when subjected to a square waveform electric field. The deflection of the collagen-filled bone shows a delayed increase, taking about one second to reach maximum deflection, while the collagen-free bone reaches its maximum almost immediately. Additionally, when electrically polarized, the bent bone cantilever gradually returns to its initial position under a constant electric field, unlike general dielectrics, which maintain a constant bend. An additional experiment using collagen-free and non-piezoelectric materials, where no streaming currents contribute to electromechanical activity, demonstrates that the flexoelectric effect in bone HA governs its behavior [69,74]. Apoptosis progression in osteocytes after bending was observed to be highest near the tip of the crack, where the strain gradient was the highest [74]. Flexoelectric fields near bone crack apices can reach several kV/m [73], similar to electrostatic fields known to induce osteocyte apoptosis. In vitro studies showed that fields as low as 1 kV/m can damage cells, and with fields over 10 kV/m causing immediate necrosis [130]. Flexoelectricity can theoretically generate such fields within tens of microns of a crack [73], with experimental evidence suggesting even more intense fields at nanoscopic distances [131].

Although the influence of piezoelectric effects of bone in the remodeling process has substantially been studied [132,133,134,135], flexoelectricity has slightly been considered. In a computational framework developed by Ganghoffer et al. (2021) [136], bone growth was predicted. However, the approach does not integrate with a finite element context, limiting its ability to simulate complex bone structures. Instead, simplified boundary value problems were studied, allowing for the derivation of analytical solutions. Recently, a numerical simulation of targeted remodeling processes (Targeted remodeling occurs in response to micro damage, and the associated death of osteocytes, which aims for the repair of the damaged bone region, whereas non-targeted remodeling is not directed towards a particular location and is regulated by changes in hormones [120,121]) in cortical bone using Isogeometric Analysis (IGA) approach considering flexoelectricity-induced osteocyte apoptosis and remodelling was proposed to investigate the process on cellular level [137]. The asymmetry in electric field distribution becomes more pronounced as specimen size decreases. In larger samples, the piezoelectric effect dominates, yielding results similar to those with zero flexoelectric coefficients. However, at smaller scales, the size-independent piezoelectric field remains constant, while the flexoelectric effect increases, eventually surpassing piezoelectricity [137]. Witt et al. (2024) [138] further developed a model of progressive crack closure using a surface growth algorithm that gradually reduces crack width and regenerates osteocyte concentration, enabling a more detailed observation of flexoelectricity’s role throughout the remodeling process, where a separation of time scales was also introduced to realistically capture the timeline of bone remodeling in numerical simulations. Incorporation of more complex signalling mechanisms, long-time diffusion of osteoclasts and osteoblasts as well as investigation of remodeling dysfunctions lined to bone diseases could be further extensions of the models. Table 2 provides an overview of major contributions regarding the manifestation of flexoelectricity in biological materials.

## 5. The Potential Biomedical Application of Flexoelectricity

Drug delivery: One of the most promising areas is micro/nanofluidic applications, where fluid-conveying nanotubes play a vital role in biological devices, particularly in smart drug delivery systems. Flexoelectric properties offer a versatile and efficient approach to develop responsive, self-powered drug delivery systems capable of precisely controlling both the amount and rate of drug release (controlled release), while also supporting localized delivery (enhancing drug targeting) with minimal invasiveness [139,140,141]. In targeted drug delivery, drugs are often encapsulated in carriers such as nanotubes, which transport and release the therapeutic agents at specific sites. Flexoelectric materials can be embedded in nanofibers or hydrogels to create platforms that respond to mechanical stimuli, allowing for on-demand drug release [140,142,143]. Mechanical deformation of these materials induces changes in surface potential, triggering the release of encapsulated drugs. Additionally, flexoelectricity significantly influences the wave dispersion characteristics in fluid-conveying cylindrical nanoshells, highlighting its broader impact on nanoscale biomedical applications [144]. Moreover, combining flexoelectric materials with other stimuli-responsive systems, such as magnetic or thermal-responsive components, could lead to multi-modal drug delivery platforms with unprecedented control over therapeutic release profiles.

Neurotransmitter: Long seen as purely chemo-electrical, the action potential is now known to involve mechanical changes in the membrane [145]. Studies showed that the flexoelectric effect of the dielectric neural membrane, triggered by the ultrasound-induced strain, is the main contributor to the linear coupling between the membrane polarization and ultrasound-induced strain for neuromodulation. This supports the effectiveness of low intensity and low frequency ultrasound sonication in modulating neural firing [146,147]. Sassaroli et al. (2016) have reviewed biophysical models and hypotheses to investigate how an acoustic stimulus might influence neuronal activity with a focus on mechanical aspects [148].

With growing demand for seamless interface between artificial devices and biological structures, flexible bioelectronics has rapidly developed in recent years [149]. Recent progress in flexible electronics has enabled the development of conformable neural interfaces that can deliver precise mechanical and electrical stimuli, adapting to the complex geometry of neural tissue to improve neuromodulation efficacy and patient comfort. Integrating flexoelectric materials into neurostimulation technologies represents a frontier in biomedical engineering, offering enhanced control over neural stimulation, potentially improving treatments for neurological disorders. Despite their promise in neurostimulation, challenges remain—particularly around biocompatibility, long-term stability, and the need for carefully designed stimulation protocols to ensure both safety and therapeutic effectiveness.

Ultrasound technologies: Implantable medical devices have transformed healthcare by enabling effective treatment of various conditions [150]. However, their reliance on batteries often necessitates replacement surgeries when power is depleted [151]. To address this, self-powered and wirelessly powered systems are emerging, with ultrasonic energy transfer to deeply implanted devices gaining significant research attention [152,153,154]. In ultrasound technologies, flexoelectric materials offer the potential to enhance transducer sensitivity by more effectively converting mechanical strain gradients into electrical signals [153].

Low-intensity, low-frequency ultrasound, based on membrane flexoelectricity, has recently gained attention as a non-invasive, cost-effective method for neuromodulation [155]. Recently, innovative tumor therapies have also emerged using ultrasound-induced flexoelectric catalysis, such as SrTiO_3_/RGD/TPP disrupting mitochondrial function, resulting in the death of tumor cells due to an inadequate energy supply [156], and MoSe_2_ nanoflowers reducing interstitial fluid pressure in photodynamic therapy [157]. These advances highlight flexoelectric catalysis as a promising strategy in nanomedicine and provide valuable insights for future applications of nanocatalysis in cancer therapy. The synergy between ultrasound’s precision (good directionality, high spatial resolution, and convenient operation) and membrane flexoelectricity is also drawing interest for treating neurodegenerative diseases like Alzheimer’s and Parkinson’s [158]. While the potential of flexoelectric materials in ultrasound imaging is convincing, challenges such as incorporating flexoelectric materials into existing ultrasound transducer designs, ensuring material compatibility and manufacturing processes, as well as achieving the desired sensitivity and performance levels in flexoelectric-based ultrasound transducers, still exist.

Tissue engineering scaffold: The use of flexoelectric materials can be seen in the field of bone remodeling-induced therapeutic strategies for bone healing and osteoporosis, such as scaffold design. A significant flexoelectric response has been confirmed in synthetic HA [73]. Therefore, materials such as polycaprolactone (PCL), polylactic acid (PLA), combined with HA, show potential as scaffolds with satisfactory flexoelectric properties to enhance bone regeneration [159]. Future research may identify naturally abundant materials with enhanced flexoelectric properties that can also withstand significant bending deformation.

Mass sensing, bio-sensors, miniature energy-storing devices used in health care, actuators and sensors in soft robotics, nano-containers for gas storage and drug delivery are other major applications of flexoelectricity from the biomedical point of view [160,161,162,163,164,165,166]. In all the above-mentioned applications, future research may focus on optimizing material properties, enhancing their biocompatibility, developing scalable fabrication techniques, and conducting clinical trials to validate the efficacy of these approaches in treating conditions.

## 6. Discussion

Flexoelectricity is a universal effect that occurs in all dielectric materials such as polymers [167,168], liquid crystals [169], biomaterials [18,73], ceramics, semiconductors [44], and perovskites under high temperatures [24], which is not limited by crystalline symmetry, depolarization temperature, and polarization. The flexoelectric effect is generally lower than the piezoelectric effect [170]. This is, perhaps, the main reason why the flexoelectric effect, upon discovery, did not attract significant interest. However, flexoelectricity has gained special attention in the last couple of decades. Aside from materials like ferroelectrics with unusually high flexoelectric coefficients, there are some situations (sometimes overlapping) that make this effect particularly important [102]: (i) when the traditional form of electromechanical coupling does not exist. For instance, biological membranes have no crystalline symmetry that would allow piezoelectricity, but changes in curvature develop polarization (flexoelectricity); (ii) when soft materials are considered, since strain gradients scale inversely with the elastic stiffness. The flexoelectric coefficients of soft materials are comparable to those of hard crystalline materials [27,28], though their elastic stiffness can be several orders of magnitude smaller than that of hard ceramics; (iii) when the feature size of the structure is small (normally around 10 nm characteristic length [18,171] and several hundreds of nanometers for soft materials [12]) since at the nanoscale, large strain gradients are easily generated to induce a strong flexoelectric response.

The flexoelectric behavior of materials is sensitive to environmental conditions such as temperature [172,173,174], pH [175,176], hydration [177,178], and the ionic environment, as these factors influence the electromechanical coupling and polarization response to strain gradients. Temperature affects molecular mobility, phase transitions, and dielectric properties of materials [17,179,180]. Near phase transition points (e.g., in lipid membranes), flexoelectric responses may increase sharply due to enhanced dielectric susceptibility [181,182]. In lipid bilayer membranes, temperature-dependent changes in membrane elasticity and dielectric behavior have been shown to significantly influence the flexoelectric effect [89]. In polymers, the temperature dependence of the flexoelectric response was shown to be closely related to relaxation processes and polymer chain mobility [174]. Most recently, Fan et al. (2025) have demonstrated temperature-sensitive flexoelectric properties in polydimethylsiloxane (PDMS), revealing that temperature variations affect the flexoelectric coefficient in a direction-dependent manner, contingent on both the polarization axis and the strain gradient orientation [183]. Increased hydration typically enhances ionic mobility and permittivity [177,184], which can in turn amplify the flexoelectric response of the material. pH plays a critical role in modulating the surface charge, protein conformation, and ionization states of biomolecules and interfaces [185]. Variations in pH can alter the surface polarization of biological membranes, thereby influencing how strain gradients induce polarization responses [186]. In polyelectrolytes or biogels, pH shifts affect their swelling behavior, which modifies local strain gradients and associated electric fields [187]. Todorov et al. (1994) investigated the influence of pH on the flexoelectric properties of lipid membranes and demonstrated that pH variations, particularly under different frequency regimes, can lead to measurable changes in the flexoelectric coefficients [175]. The ionic environment—including ion concentration, strength, and type—significantly influences polarization dynamics [188,189], which in turn affects the manifestation of flexoelectric effects. This is because the displacement of ions in response to non-uniform mechanical strain directly contributes to the material’s polarization behavior [190]. The ionic conductivity serves as a critical factor in enhancing the energy-capturing efficiency of flexoelectric energy harvesters [160,191], as the limited mobility of ions at higher frequencies results in significantly lower flexoelectric coefficients compared to those measured at lower frequencies. Inspired by ion currents and ionic polarization in living organisms, Jia et al. (2024) reported that the pronounced flexoelectricity observed in soft hydrogels stems from ion polarization due to differing cation and anion transport rates under bending-induced strain gradients [160]. They further demonstrated that both the magnitude and polarity of the flexoelectric response can be tuned by selecting specific cation–anion pairs and adjusting the polymer chain network within the hydrogel (enhanced flexoelectric coefficient from 1160 $\mu Cm^{-1}$ to 2340$\;\mu Cm^{-1}$). In a comprehensive review, Petrov (2002) [57] highlighted how variations in ionic conditions modulate the flexoelectric properties of biological systems, emphasizing that changes in the electrostatic environment can strongly impact the polarization response [57]. These effects are relevant when designing biosensors or bioelectronic interfaces using flexoelectric materials.

Many efforts have been made to identify the flexoelectric effect both experimentally and theoretically. Several reviews have already been published on flexoelectricity in solids [29,31,117,192], where the large picture of the field is found. However, the contradictory theoretical and experimental results have led to a limited understanding of flexoelectricity [29] and make their comparison a challenging task. The flexoelectric effect is known to be susceptible to structural defects such as grain boundaries [193], doping, and dislocations [194]. Therefore, unexpected micro-scale heterogeneity in materials is the reason for the discrepancy observed between experimental values and their related theoretical calculations [195]. The strain, strain gradient, and polarization, defining the state of a material, are often not uniform throughout the material. As a result, examining the pointwise state of these variables can be computationally expensive and impractical. While MD modeling and first-principles calculations can predict the pointwise state of a system, they face limitations related to time and size scales [16]. Currently, the temperature dependence of flexoelectric response can be said to be often close to that of the dielectric constant, compatible with the theory [17,20], and the orders of magnitude of the flexoelectric coefficients in crystals are consistent with the rough estimate by Kogan [21]. However, a more quantitative comparison with the theory is not currently possible. The macroscopic characterization provides information on the static or dynamic bulk flexoelectric response and/or the contribution of the surface piezoelectricity [29]. Therefore, the interpretation of the data is also challenging, especially considering the potential contribution of surface piezoelectricity, for which a quantitative theory is not yet available.

Many advancements have occurred in the experimental identification of flexoelectricty, however, the limitations do still exist. For instance, generating submicrometric oscillations to induce small strain gradients is impossible in some direct methods such as bending tests using dynamical mechanical analyser resulting in a rather small window for measurements [45]. Also, the mechanical resilience of samples (whether they break by increasing the amplitude) directly limits the achievable oscillation amplitude. The maximum operational frequency is another important factor since low frequencies might cause issues like not reaching the limit of detection of the signal [35]. The fact that a relatively large sample (on a millimetre scale) is required for such a microscale effect could be restrictive, but nano-indentation techniques allow testing materials, where submillimetre oscillations and relatively small strain fields are required [35]. Carefully designing experimental measurements is crucial, as electromechanical coupling can arise from various intrinsic and extrinsic mechanisms. This allows for the identification of the specific mechanism responsible for the observed electromechanical coupling processes [1]. From a computational point of view, the flexoelectric effect in the context of small strains [25,196] has been investigated. To address the incorporation of gradient contributions in the energy function, different approaches have been used such as B-spline immersed boundary approaches [197], mixed finite elements [198,199], meshfree methods [196], micromorphic approaches [200], $C^{0}$ interior penalty methods [201], or isogeometric analysis (IGA) [137,138,202,203,204], which all have their own cons and pros and interested readers are referred to the references for deep understanding as computational details of flexoelectricity is outside the scope of the current study.

Flexoelectricity has been observed in cells and cell membranes, hearing mechanisms, and bone remodeling near the crack [74,88,104,105]. The studies reviewed in the current work present a combination of theoretical models and experimental insights into the role of flexoelectricity, which has been discovered in healthy biological materials. It is worth noting that healthy and unhealthy (tumor) tissues exhibit distinct electricomechanical properties, primarily due to differences in cellular structure, the composition and organization of the ECM, metabolism, and electrolyte concentrations [205]. Mechanically, tumors typically present elevated interstitial fluid pressure [206] and are stiffer than normal tissues, largely due to the excessive deposition of components such as collagen, proteoglycans, and fibronectin within the tumor stroma, which promote tumor growth and invasion [207]. Despite the increased stiffness of the tumor mass, cancer cells themselves are generally more deformable, enabling them to squeeze through confined spaces during invasion and metastasis [207]. Electrically, tumor tissues often exhibit higher conductivity and permittivity, attributed to increased water content, ionic concentrations, and altered cell membrane properties [208,209,210]. These changes result in lower electrical impedance compared to healthy tissues [211]. Additionally, tumor cells frequently display disrupted ionic balances, reduced transmembrane potential, and altered intra- and extracellular pH levels, contributing to the acidic nature of the tumor microenvironment [211]. Understanding these electromechanical differences—and the associated changes in cellular and ECM structure and function—is essential for developing novel therapies that target the tumor microenvironment and improve treatment outcomes [212].

**Figure 2:**
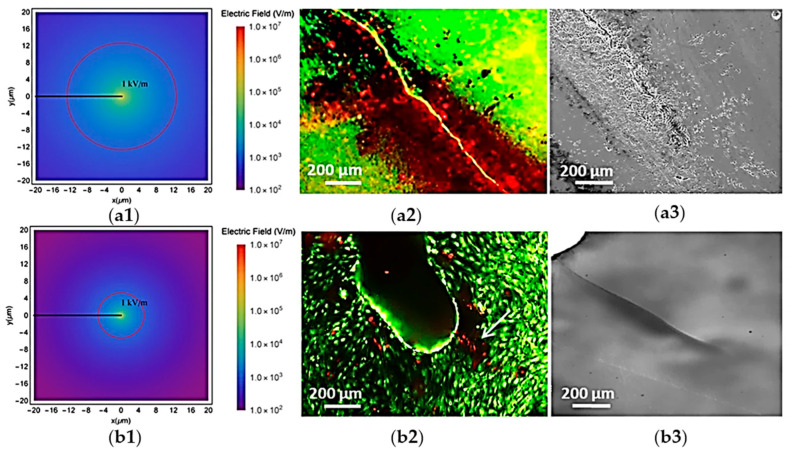
Flexoelectricity around bone microcracks in TiO_2_ and HA. Distribution of the lexoelectric field around a microcrack in HA (**a1**) and TiO_2_ (**b1**). Stained images of osteocytes cultured in HA (**a2**) and TiO_2_ (**b2**) directly after stimulation, where the white arrow indicates the localization of the cracks. The red circle marks the boundary of the 1 kV/m electric field region—the minimum field strength known to cause cellular damage. Microcracks images taken before cell culture in HA (**a3**) and TiO_2_ (**b3**) substrates (adapted from Núñez-Toldrà et al., 2020 [74]).

Among biological materials exhibiting flexoelectric effects, we focused on bone more than others since both experimental and computational aspects of the role of flexoelectricity have been taken into account, which might provide us with a clearer insight. Many factors in the surrounding environment influence bone growth, healing, and health. One of the most studied environmental factors is mechanical stimulation, which has been a key focus of research for years [213,214,215]. Recently, the discovery of bone electromechanical effects such as piezoelectricity [107,109,216], streaming potentials [217,218], and flexoelectricity [73] has opened a new area in bone mechanics that can potentially lead to many advancements in clinical applications. Bone remodeling near the crack, in particular, has received huge benefit from the flexoelectric effect, as evidenced by experimental studies performed in this regard [73,74]. The mere presence of the crack does not cause apoptosis; instead, the crack must be mechanically stimulated for cells to die [73,74]. Comparison of the apoptotic effect in cracks induced by mechanical stimulation on single crystal substrates of titania dioxide (TiO_2_), which, like HA, only exhibits flexoelectricity, revealed notable differences. Unlike HA, TiO_2_ differs in its theoretical spatial distribution of flexoelectric fields, leading to a distinct apoptotic response. The results showed that the TiO_2_ apoptotic effect of crack flexoelectricity is confined to cells located directly near the crack (Figure 2(b2)). However, the apoptotic region observed in HA was significantly larger (Figure 2(a2)). The extension of the “damage zone” is justified by the differing crystal morphology of TiO_2_ crystals from that of the HA. In HA, cell apoptosis occurs at distances beyond those predicted theoretically (Figure 2(a1)), possibly due to additional irregular, branched microcracks and surrounding mechanical damage near the main crack (Figure 2(a3)). In contrast, a clean, unbranched cleavage was shown in the crack in the TiO_2_ single crystal (Figure 2(b3)). Therefore, it is consistent with the ideal case simulated by the calculations to have a smaller theoretical radius of flexoelectricity. Both the material properties and the distinct crack morphology contribute to the overall extent of the apoptotic region.

Bone is also a hierarchical tissue with the following identified levels: (i) the macrostructural level of entire bone, (ii) the meso scale cortical bone with randomly organized osteons, (iii) the microstructural level of a single osteon with concentric layers of lamellae (up to hundreds microns), (iv) the sub-microstructural level of a single lamellae containing lacunae (tenths to hundreds microns), and (v) the nanostructural level (few up to several hundred nanometers) [217,218]. Flexoelectricity is a size-dependent effect and other size-dependent phenomena, such as strain gradient elasticity, surface piezoelectricity and flexoelectricity, dynamic flexoelectricity usually coexist [16]-Therefore, a comprehensive study should investigate at which scale each electromechanical effect occurs. Regardless of the bone studies taking the influence of individual electromechanical effects into account, to the best of the authors’ knowledge, there is still no study to investigate the couple effects and evaluate how flexoelectricity along with piezoelectricity can enhance bone remodeling and healing. Therefore, computational multiscale modeling serves as a promising approach to take both effects into account. Furthermore, it is important to distinguish whether the respective bone remodeling processes (with separate electromechanical effects) happen in cortical or in cancellous bone, since these processes differ [120]. Therefore, coupling the electromechanical effects happening in the right type of bone will lead to a more realistic model for bone remodeling. Having in mind that cortical bone fails at strains of approximately 2% [219], a small-strain theory is deemed appropriate for modeling cortical bone, even though under certain conditions, bone may exhibit larger strains. However, for soft tissues, which are sensitive to electromechanical signals (with the cell membrane exhibiting flexoelectric effects [220,221]) and undergo large strains, extending the modeling framework to incorporate coupled electromechanical phenomena within a large strain formalism will be a crucial direction for future development.

The studies presented in this review provide compelling evidence for the role of flexoelectricity in biological materials. However, in fields like bioelectromechanics and mechanobiology, ensuring physiological relevance is crucial for improving the translational value of research. It is important to note that, to date, flexoelectricity in biological tissues has not been fully realized under realistic physiological conditions. Specifically, the experimental setup might introduce non-physiological conditions that alter the outcomes. While the findings contribute significantly to the field, their physiological relevance remains limited. The idea that physiological realism is essential for interpreting biomechanical or bioelectrical responses in living tissues cannot be overstated [222]. Therefore, while these studies offer valuable insights, further work is needed to replicate and understand these effects in authentic biological contexts. Examples of current limitations that hinder the full capture of physiological relevance for these materials are as follows:

Cell and bio-membranes: While DIBs provide a highly controlled and tunable platform to study lipid bilayers, they present several limitations in mimicking the complex environment of biological membranes. One of the primary drawbacks is the absence of native membrane asymmetry and curvature, which are critical to the function of bio-membranes in vivo. DIBs typically use symmetric lipid compositions and flat bilayer geometries, whereas physiological membranes are highly dynamic, curved, and compositionally heterogeneous, with distinct lipid distributions between the inner and outer leaflets [223]. Additionally, DIBs often lack membrane-associated proteins, cytoskeletal elements, and ECM components, all of which are integral to native membrane structure and function [224]. Furthermore, while the oil phase used in DIBs is necessary for stabilizing the droplet interface, it introduces a non-physiological environment that can alter membrane properties such as lipid packing, fluidity, and electrical characteristics [224]. Furthermore, the mechanical environment in DIB setups does not replicate the complex biomechanical cues and osmotic gradients present in tissues, which are essential for accurate mechanotransduction studies. These limitations must be considered when interpreting results from DIB experiments and assessing their physiological relevance. Another significant limitation is the lack of appropriate detection techniques to quantify molecular transport within DIBs, which restricts their application in more diverse research fields. Addressing these challenges is crucial for enhancing the biomimetic nature of DIBs and improving their relevance for more complex biological studies [224]. Bioelectronic devices made from 2D droplet networks that interact with living systems must closely align with the soft and flexible nature of biological tissues to realistically replicate their mechanical properties. This alignment is crucial to ensure that the devices can seamlessly integrate with living tissues without causing undue stress or damage. By matching tissue mechanics, such devices reduce inflammation and scarring, improving biocompatibility and enabling long-term use for effective bioelectronic interfaces [97]. Most DIB applications rely on static architectures, which limit their ability to adapt over time. In contrast, living organisms continuously reshape and adapt their membranes to maintain functionality, a dynamic process that has not yet been replicated in DIB systems [98]. While efforts to improve the adaptability of DIB networks—such as through selective droplet coalescence [98]—show promise, these approaches still face significant challenges in mimicking the dynamic and self-repairing nature of biological systems. Achieving a more dynamic, self-regenerating architecture would be crucial for enhancing the long-term performance and biocompatibility of DIB-based bioelectronic devices.

Hearing system: The cochlea is a complex, fluid-filled organ with finely tuned mechanical and electrical gradients [225]. Hearing depends on active processes like electromotility, ion channel dynamics, and membrane potential fluctuations [225]. Yet, most studies on flexoelectricity focus on isolated outer hair cell membranes or simplified bilayers, neglecting the coupled behavior of cells, fluids, and structural components [75,103,226]. These models often overlook key in vivo features such as ionic gradients, membrane asymmetry, and mechanical feedback loops, limiting their physiological relevance. This separation obscures how flexoelectric responses might be modulated by biological activity and vice versa. Moreover, these models frequently rely on simplified assumptions, such as smooth membrane surfaces, pure bending deformation, and a linear relationship between membrane polarization and mechanical strain [71,103]. The parameters used in the modeling—such as flexoelectric coefficients, dielectric constants, and membrane moduli—are often estimated from non-auditory or synthetic systems, which may not faithfully represent the biophysical properties of outer hair cell membranes. Boundary conditions, such as fluid–structure interactions in the cochlear duct, are commonly oversimplified or excluded entirely, further reducing model realism. Direct measurement of flexoelectricity at the nanometer scale remains technically difficult; techniques like atomic force microscopy (AFM) lack the resolution to capture real-time outer hair cell dynamics. Consequently, current findings, based largely on indirect inference or idealized models, may not accurately reflect the in vivo function of flexoelectricity in hearing [227].

Bone: Flexoelectricity is a nanoscale effect, yet experiments often rely on microscale samples, which may overlook localized effects [73,74]. Bone is inherently heterogeneous, anisotropic, and porous, which complicates accurate, artifact-free electrical measurements. Electrodes used in experiments may induce local polarization, leading to misinterpretation of flexoelectric signals. Furthermore, in vitro studies do not replicate true in vivo conditions. Bone samples used in these studies are often dehydrated, demineralized, or highly processed (e.g., bovine, cortical-only, or synthetic analogs), and HA samples used may differ in porosity compared to natural bone. These samples lack essential features such as hydration and collagen-mineral interactions, which are critical to bone’s electromechanical and adaptive behaviors. In vitro, flexoelectricity may be sufficient to account for the apoptotic effects of microcracks without the need for piezoelectric collagen or streaming currents [73,74]. However, despite the potential, measuring flexoelectricity in vitro remains challenging. Advancing technology is required to place very small electrodes at the micro/nanoscale, potentially enabling direct in vivo measurement of flexoelectric fields, especially since other mechanisms may also contribute in vivo. Experimental setups typically apply idealized mechanical deformations (e.g., uniform bending and high strain gradients) to elicit measurable flexoelectric signals, conditions that often do not reflect the complexity of real biological environments. Theoretical models of bone flexoelectricity often rely on experimental findings that do not capture the full physiological environment of bone tissue. Computational models assume simplifications in material modeling, use simplified geometries, and neglect fluid–solid interactions (e.g., poroelasticity), which are essential for accurately simulating its biological function [137,138]. Table 3 summarizes the key limitations of current experimental models in capturing the physiologically relevant flexoelectric behavior of these biological tissues. Therefore, at the basic research level, experimental identification of direct and converse flexoelectric effect in biological materials and investigation of whether the theoretical framework can explain what observed experimentally (in physiological conditions) or vice versa will be a point of further research as an effort to find out why the flexoelectric coefficients of some materials are beyond the theoretical value.

Identifying methods to induce large strain gradients—beyond relying solely on size effects—would represent a significant advancement in experimental research. Investigation of characteristic properties of flexoelectric materials and coupled effects is always an open area. The significance of novel coupling modes—such as the interaction between polarization fields and strain gradients enabled by flexoelectricity—is often underestimated. Advancing research in these areas could clearly differentiate flexoelectricity from piezoelectricity and greatly benefit the development of smart biomedical devices.

## 7. Conclusions and Outlook

In summary, this review explores the phenomenon of electromechanical coupling of materials, with a particular focus on flexoelectricity. It highlights the growing interest in flexoelectric materials over the past few decades, despite the concept itself being introduced as early as the 1960s. The review outlines key reasons for this renewed attention, especially in comparison to more established piezoelectric materials.

The discussion then centers on the experimental identification of flexoelectricity and its limitations, with particular emphasis on the two most commonly employed techniques: cantilever bending and truncated pyramid indentation, widely used to measure the flexoelectric coefficient in various materials, as summarized in Table 1. Theoretical foundations of flexoelectricity were also briefly reviewed; however, the emphasis is placed on reported results and their implications, rather than in-depth mathematical formulations. While not exhaustive, this review aims to guide interested readers toward relevant theoretical references. Looking ahead, the field of flexoelectricity holds substantial promise for advancing both fundamental understanding and practical applications. A clear microscopic underpinning of flexoelectricity in soft materials is still lacking, although the fundamentals of flexoelectricity are relatively well-understood [29]. Therefore, a provoking future direction is enhancing polarization in soft materials (hydrogel and biomembranes), where larger strain gradients and deformations are more feasible, offering strong flexoelectric responses. Theoretically, the theories of diffusion and flexoelectricity can be combined for soft materials to account for the interplay between flexoelectricity and ion conductivity [161,228].

A dedicated section addresses flexoelectricity in biological materials, with a focus on cells, bio-membranes, hearing mechanisms, bone and its remodeling. The authors aim to highlight computational and experimental studies that demonstrate the presence of flexoelectric effects in biological systems, thereby underscoring the relevance of this phenomenon to emerging biomedical research. However, it is worth reminding that most of the original research works on cell and bio-membranes were focused on computational aspects, which brings a future direction toward designing appropriate experimental setups to measure and observe the effect. A better understanding of the mechanism can unlock new ways to influence tissue behavior using mechanical forces and electrical stimuli. The study of flexoelectricity in biological materials is an evolving area of research.

The review covered the clinical and biomedical potential of flexoelectricity, including its applications. As the field develops, it holds the potential to revolutionize our understanding of mechanotransduction and offer new therapeutic strategies in regenerative medicine and scaffold design for the tissues that particularly experience significant strain gradients, such as bone. Future work is also expected to focus on the design of biocompatible flexoelectric materials with tunable properties tailored for specific biomedical functions, such as fluid-conveying flexoelectric nanotubes, smart scaffolds that respond to mechanical stimuli, or self-powered biosensors for real-time physiological monitoring. A deeper investigation into the cellular and molecular mechanisms influenced by electromechanical coupling could open new avenues in tissue engineering, particularly in guiding stem cell differentiation and regenerative processes. Flexoelectric materials could improve the detection capabilities of ultrasound transducers, leading to higher-resolution images for many biomedical applications, advancing medical diagnostics. Their integration into flexible transducers allows better conformity to complex body surfaces, improving both patient comfort and diagnostic accuracy. Additionally, emerging micro- and nanoscale fabrication techniques will likely enable the integration of flexoelectric elements into implantable devices, neurotransmitters, ultrasound technologies, biosensing, miniature energy storage devices, drug delivery systems, and lab (organ)-on-a-chip platforms, where mechanical and electrical cues mimic physiological conditions for more accurate drug testing. The development of computational models to predict flexoelectric behavior in complex biological environments, coupled with multiscale experimental validation, will be critical in bridging the gap between material design and clinical implementation. Overall, flexoelectricity represents a dynamic frontier with vast potential to impact next-generation healthcare technologies.

## Figures and Tables

**Table 1:** Measured flexoelectric coefficient values for various flexoelectric materials.

Material	Measurement Method	Flexoelectric Coefficient [C/m]	References
(K,Na,Li)(Nb,Sb)O_3_ (KNNLS)	Three-point bending	10^−6^	[42]
Liquid crystal materials	Pure bend and splay	10^−8^	[43]
Barium Titanate (BaTiO_3_)	Cantilever bending	1 × 10^−6^	[44]
Barium Strontinum Titanate ((Ba_(1–x)_ Sr_x_)TiO_3_)	Pyramid compression	1^−10^ × 10^−9^	[2,31]
Strontium Titanate (SrTiO_3_)	Dynamic mechanical analyzer, three-point bending, cantilever beam	1–10 × 10^−9^	[35,45,46]
Polyvinylidene fluoride (PVDF)	Cantilever beam-based approach	1.1 (±0.1) × 10^−8^	[47]
Graphene	Molecular dynamics simulations	1.53 × 10^−16^–5.18 × 10^−13^	[48]
Silicene (flat)	Molecular dynamics simulations	1.04 × 10^−14^–1.91 × 10^−14^	[48]
Silicene	Molecular dynamics simulations	2.7 × 10^−12^–2.94 × 10^−14^	[48]
Boron nitride (BN)	Molecular dynamics simulations	1.46 × 10^−12^–3.06 × 10^−14^	[48]
Amorphous HfO_2_	Laser Doppler Vibrometer-based approach	105 (±10) × 10^−12^	[49]
Oriented PET	Cantilever bending	9.9 (±0.4) × 10^−9^	[40]
Polyethylene	Cantilever bending	5.8 (±1.1) × 10^−9^	[40]
Epoxy	Cantilever bending	2.9 (±0.3) × 10^−9^	[40]
BST/Ni_0.8_Zn_0.2_Fe_2_O_4_	Conventional pure bending	128 × 10^−6^	[50]
Single crystals of Titanium Dioxide (TiO_2_)	Three-point bending	1–10 × 10^−9^	[46]
Halides (XPbBr_3_ and XPbCl_3_)	Oscillatory bending induced by piezoelectric actuator	3 × 10^−5^	[6]
Molybdenum disulfide (MoS_2_)	Piezoresponse Force Microscopy	0.1 × 10^−9^	[51]
	Molecular dynamics simulations	1.6–9.6 × 10^−12^	[48]
Zinc/aluminum-layered double hydroxides nanosheets	Bending test	1.8 (±0.35) × 10^−6^	[52]
(Bi_1.5_Zn_0.5_)(Zn_0.5_Nb_1.5_)O_7_/Ag	Beam bending method	1.7 × 10^−7^	[53]
NaNbO_3_ nanotube/epoxy composite	Cantilever bending	2.77 × 10^−8^	[54]

**Table 2:** A summary of key insights into flexoelectric phenomena observed in biological materials.

Biological Material	Key Findings	References
Cell	Development of a coupled 2D model of biological cells with piezoelectric and nonlocal flexoelectric properties of their organelles Flexoelectricity generates electric fields up to four orders of magnitude stronger than those from piezoelectric effects alone.	[82]
	Developed models to investigate separate electromechanical response from Microtubule	[83,84,85]
	The role of cell structure and organelles in flexoelectric behavior of biological cells	[70]
Bio-membrane	The microscopic groundwork of flexoelectricity were established by Petrov for biological membranes in a series of founding studies	[57,58,93,94]
	Flexoelectric effects are relevant to study ion channels, thermal fluctuations, and equilibrium shape of the vesicle.	[12,89,90,91]
	Flexoelectricity in membranes results from the interaction between the electric field and the molecular multipoles, leading to an inhomogeneous electric force across the membrane. Effects of ions, PH, external potentials are a simple physicochemical phenomenon in essence, but spatial and temporal interconnection of them occurring simultaneously and locally in different regions of the membrane regulate very complex electromotility.	[92]
	Development of a continuum model to assess the electrostatic contribution to membrane instability and bending rigidity using the Poisson-Boltzmann equation	[95]
	A simple model of counter-ion absorption was developed to investigate voltage-induced bending and electromechanical coupling in lipid bilayers. Lower flexoelectric coefficients in comparison with experimental data were predicted.	[57,96]
	Use of droplet interface bilayer (DIB) technology to study bio-membrane flexoelectricity This technique can be used to investigate mechanotransduction and the interfacial properties of unsupported liquid biomimetic membranes.	[97,98]
	Bio-membranes with smaller radii (higher curvature) exhibit a stronger flexoelectric response.	[58]
	Flexoelectricity in bio-membrane can drive the activation of tension-gated channels within the membrane.	[99,100,101]
	Investigation of the role of flexoelectricity in modifying the entropic force between two fluctuating fluid membranes using a variational perturbative approximation	[88]
Hearing system	Petrov (1994) was the first to propose that direct flexoelectricity might trigger mechanotransduction in auditory hair cells	[105]
	There is substantial evidence to show flexoelectricity as the major mechanism behind outer hair cell electromotility.	[13,71,75,92,102]
	Several works have indicated flexoelectricity as an important electromechanical coupling in hearing system.	[103,104]
	Stereocilia are fast flexoelectric motors and highly efficient to capture the energy in the extracellular electro-chemical potential of the inner ear to generate mechanical power output.	[13]
	Development of a physical model of hair bundles by incorporating flexoelectricity and a physics-based nonlinear dynamical model. Due to flexoelectricity, certain combinations of inner surface charge density and membrane bending stiffness can cause hair bundle oscillations to become unstable, enabling the amplification of weak acoustic signals. Increasing the bending modulus can stabilize the system, potentially impairing the hair bundle’s ability to amplify external stimuli. Flexoelectricity plays a key role in inducing the Hopf bifurcation state, which is believed to underlie several highly nonlinear aspects of the hearing mechanism.	[75]
Bone	Bone’s hierarchical structure gives rise to various deformation gradients that promote flexoelectricity, particularly near microcracks.	[76,113]
	A very first theoretical analysis on the potential role of strain gradients in bone	[114]
	Information on bending-induced polarization in bones, mistakenly attributed to collagen	[115]
	Experimental confirmation on the presence of substantial strain gradient fields in bone.	[116]
	Comparison of bone and pure HA flexoelectricity using the cantilever system to clarify the origin of bone flexoelectricity. HA governs the flexoelectric behavior.	[69,73,74]
	Near bone cracks, flexoelectricity is theoretically highest where there is neither collagen nor streaming potentials. Flexoelectric fields near bone crack apices can reach several kV/m. Flexoelectricity plays two different roles in bone remodelling near cracks: apoptotic triggering of the repair protocol, and electro-stimulating the bone-building activity of osteoblasts.	[73,74]
	Computational frameworks for bone growth and remodeling In larger samples, the piezoelectric effect dominates. However, at smaller scales, the flexoelectric effect increases, eventually surpassing piezoelectricity	[136,137,138]

**Table 3:** Assessing the physiological relevance of experimental electromechanical models in the cell and bio-membrane, the hearing system, and the bone.

Tissues	Limitations Posed by Experimental Conditions	Comparison to In Vivo Physiological Conditions	References
Cell and bio-membrane	Limitations with DIB: Use of symmetric lipid compositions and flat bilayer geometries	Physiological membranes are highly dynamic, curved, and compositionally heterogeneous, with distinct lipid distributions between the inner and outer leaflets.	[97,98,223,224]
	lack of membrane-associated proteins, cytoskeletal elements, and ECM components	Membrane-associated components are integral to native membrane structure and function.	
	Oil phase introduces a non-physiological environment altering membrane properties.	Biological membranes form in aqueous environments, without an interfacial oil phase.	
	Mechanical environment in DIB setups do not replicate the complex biomechanical cues and osmotic gradients present in tissues.	Membrane tension is often passively set by droplet size or manipulated externally, not dynamically regulated as in cells. Osmotic gradients can be imposed manually, but they are not sustained or physiologically complex. Shear stress (e.g., blood flow) and substrate stiffness/ECM interactions are present in real bio-membrane.	
	Most DIB applications rely on static architectures.	In contrast, living organisms continuously reshape and adapt their membranes to maintain functionality	
Hearing system	Focusing on isolated outer hair cell membranes or simplified bilayers	Cochlea is a complex organ with finely tuned mechanical and electrical gradients.	[71,75,103,225,226,227]
	Neglecting the coupled behavior of cells, fluids, and structural components	Cochlea is a fluid-filled organ.	
	Ignoring key in vivo features	The electromechanical function of the auditory system relies critically on ionic gradients, lipid membrane asymmetry, and tightly regulated mechanical feedback loops.	
Bone	Flexoelectricity has been measured often on microscale samples.	Flexoelectricity is a nanoscale effect, requiring very small electrodes at the micro/nanoscale, enabling direct in vivo measurement of flexoelectricity.	[73,74]
	Electrodes used in experiments may induce local polarization, leading to misinterpretation of flexoelectric signals.	Bone is inherently heterogeneous, anisotropic, and porous, which require accurate, artifact-free electrical measurements.	
	In vitro studies do not replicate true in vivo conditions.	In vivo, other mechanisms may also contribute to flexoelectric fields.	
	Dehydrated, demineralized, or highly processed samples have been used	Hydration and collagen-mineral interactions are essential features in bone’s electromechanical and adaptive behaviors. Porosity of own-made HA samples differs from reality.	
	Use of idealized mechanical deformations	uniform bending and high strain gradients do not reflect the complexity of real bone environments	

## Data Availability

No new data were generated or analyzed in this study. Data sharing is not applicable to this article.
